# Peak frequency mapping to differentiate the sinus node and superior vena cava during atrial fibrillation

**DOI:** 10.1016/j.hrcr.2025.07.008

**Published:** 2025-07-12

**Authors:** Masataka Narita, Hitoshi Mori, Kazuhisa Matsumoto, Yoshifumi Ikeda, Ritsushi Kato

**Affiliations:** Department of Cardiology, Saitama Medical University International Medical Center, Hidaka, Saitama, Japan

**Keywords:** Immediate recurrence of atrial fibrillation, Superior vena cava isolation, High-density mapping, Omnipolar mapping, Peak frequency, Sinus node

**Key Teaching Points**

• The superior vena cava (SVC) is a common source of immediate recurrence of atrial fibrillation, and electrical isolation of the SVC is an important therapeutic strategy. However, SVC isolation carries a risk of complications, including sinus node injury.
• To date, no studies have reported the effectiveness of 3-dimensional mapping in identifying the sinus node during atrial fibrillation.
• In our case, we could visualize the SVC-right atrium junction during atrial fibrillation by gradually increasing the cutoff frequency on the emphasis map.

## Introduction

Treating the immediate recurrence of atrial fibrillation (IRAF) triggers can improve the success rate of atrial fibrillation (AF) management. However, identifying and effectively targeting those triggers remain challenging.^1^ The superior vena cava (SVC) is a common origin of IRAF, and its electrical isolation is an important therapeutic strategy. However, SVC isolations are sometimes associated with complications such as sinus node injury (SNI). Detailed mapping to identify the sinus node area before ablation is useful to avoid SNI. Nevertheless, in cases where sinus rhythm cannot be maintained owing to IRAFs, it becomes difficult to localize the SVC-right atrium (RA) junction.

This report presents a case where the emphasis map, using the peak frequency annotation algorithm in the EnSite X EP System (Abbott, Abbott Park, IL), proved to be a useful tool in the management of IRAFs originating from the SVC.

## Case report

A 54-year-old man, noted to have AF during a routine health checkup 2 years earlier, visited a local clinic and was found to have recurrent episodes of AF. He was diagnosed as having paroxysmal AF that was resistant to antiarrhythmic drug therapy at a relatively young age. He was referred to our hospital for catheter ablation. Four months before the current admission, he underwent pulmonary vein (PV) isolation and cavotricuspid isthmus ablation. However, he experienced recurrent AF and was admitted for a second ablation session.

After deep sedation, vascular access was obtained via the femoral vein. A BeeAT catheter (Nihon Kohden Corporation, Tokyo, Japan) was positioned into the coronary sinus. A transseptal puncture was then performed. HD grid (Abbott) and TactiFlex catheters (Abbott) were positioned in the left atrium. Cardioversion was attempted to terminate AF; however, AF recurred immediately, and sinus rhythm could not be maintained. The earliest site of the IRAF was recorded on the proximal electrodes of the coronary sinus catheter, suggesting septal, RA, and SVC origin. After positioning the ablation catheter in the SVC, cardioversion was repeated, which revealed an IRAF originating from the SVC. Based on this finding, the arrhythmia was diagnosed as an SVC-origin IRAF, and an SVC isolation was subsequently planned.

**Figure 1:**
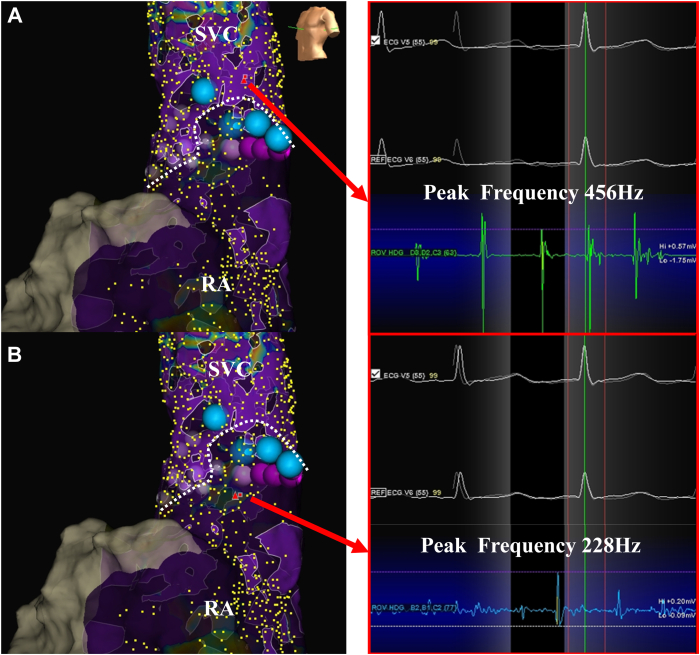
High-density mapping during AF. High-density mapping during AF revealed a sharper annotated peak frequency in the SVC (**A**) than in the RA (**B**). When the peak frequency map and emphasis map were applied with a threshold of ≥350 Hz, the emphasis map highlighted the SVC region, whereas no emphasis was observed in the RA. Consequently, the SVC-RA junction was clearly delineated (*dotted line*). The *blue tags* correspond to the region of lesions for the emphasis map–guided ablation. An SVC isolation was successfully created by ablation in the highlighted area, which corresponded to the SVC region. AF = atrial fibrillation; RA = right atrium; SVC = superior vena cava.

We attempted to map the sinus node area by placing the HD grid catheter at the SVC-RA junction. However, the IRAF immediately occurred, and the sinus mapping could not be performed. Therefore, detailed SVC-RA mapping was created using an HD grid catheter under AF before the SVC isolation. Phrenic nerve capture sites identified with the ablation catheter were recorded, and ablation was subsequently performed in the posterior and septal regions based on the anatomic information using a power setting of 50 W for 8 seconds, while avoiding both SNI and phrenic nerve injury. However, the SVC isolation could not be achieved. Given that the mapping was performed during AF, identifying the sinus node area using an activation map was impossible. When the peak frequency map and emphasis map were applied with a threshold of ≥350 Hz, the emphasis map highlighted the SVC region, whereas the RA was not visualized on the emphasis map. Consequently, the SVC-RA junction was clearly delineated (Figure 1).

**Figure 2:**
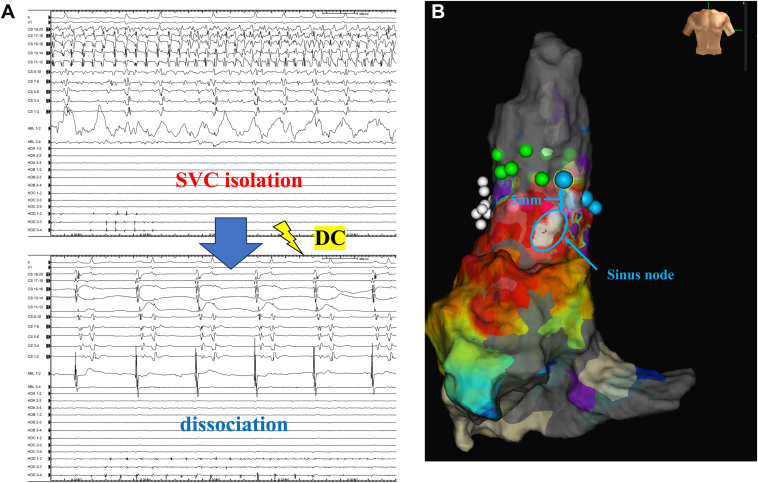
The SVC was successfully isolated electrically. After direct current cardioversion, an electrical dissociation was observed within the SVC (**A**). After touch-up ablation, electrical isolation was confirmed using a 3-dimensional map (**B**). **B**: The *blue tags* were the region of lesions for the emphasis map–guided ablation, and the *green tags* were the touch-up site. *White tags* indicate locations where diaphragmatic stimulation was confirmed. The distance between the sinus node (*blue circle*) and the *blue tags* is 5 mm. SVC = superior vena cava.

**Figure 3:**
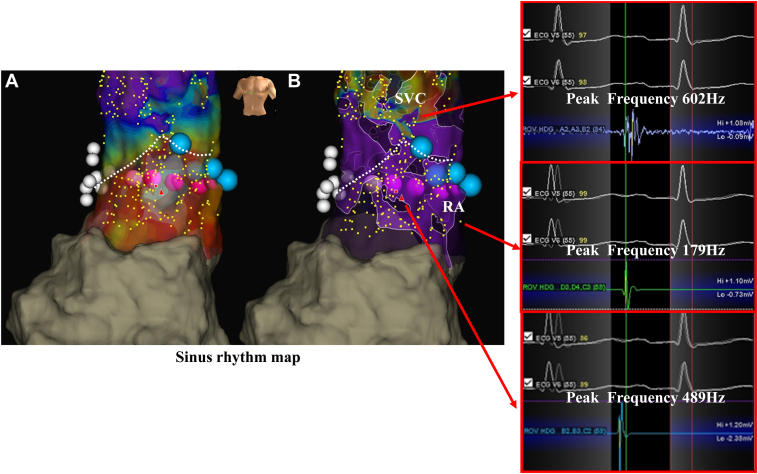
Activation map (**A**) and emphasis map (**B**) during sinus rhythm revealed frequencies of 489 Hz in the RA and 602 Hz in the SVC, which were higher than those observed during AF. The *blue tags* are the region of lesions for the emphasis map–guided ablation. The *white tags* mark sites of confirmed diaphragmatic stimulation, and the *dotted line* represents the SVC-RA junction. AF = atrial fibrillation; RA = right atrium; SVC = superior vena cava.

When ablation was performed on the anterior aspect of the SVC at the highlighted zone, corresponding to the SVC region, the electrical isolation of the SVC was successfully achieved. After direct current cardioversion, electrical dissociation was observed within the SVC (Figure 2A). During the waiting period, reconduction of the SVC was observed. Repeat mapping under sinus rhythm revealed reconduction through a gap (Figure 3). Additional ablation was performed at the identified gap, and after touch-up ablation, the electrical isolation was confirmed using a 3-dimensional map (Figure 2B). No dormant conduction was observed, and the procedure was concluded. The patient was discharged from the hospital without any complications after the procedure.

## Discussion

The SVC is considered to be the most common AF trigger from extra-PV, which harbors 26%–30% of non-PV foci.^2^ For the SVC isolation, an accurate localization of the sinus node using activation mapping is crucial for avoiding SNI. SNI occurred in 4.5% of cases with SVC isolation.^3^ The precise anatomic location of the sinus node often remains unclear in patients with IRAFs, even when voltage mapping is used. No studies have reported on the effectiveness of 3-dimensional mapping in identifying the sinus node during AF.

The EnSite OT near-field algorithm automatically quantifies and annotates the intracardiac electrogram based on the electrogram frequency.^4^ The peak frequency provides a quantitative measure for evaluating the sharpness of morphologic features within the intracardiac electrogram, thereby aiding in the differentiation between near- and far-field components.^4^ Glashan et al^5^, ^6^, ^7^ demonstrated that local unipolar and bipolar electrogram amplitudes correlate with the distribution of fibrosis, myocardial muscle volume, and wall thickness. Furthermore, Takamiya et al^4^ reported that, using the EnSite X system in healthy hearts, the median peak frequency was significantly higher in the SVC than in the RA, whereas the median maximum voltage was significantly lower in the SVC than in the RA. Low-amplitude potentials observed in the SVC indicate its thin myocardial wall. In such thin-walled structures, electrical conduction from myocardial fibers located immediately beneath the surface can be recorded, whereas signals from deeper myocardial layers are less likely to be detected. Consequently, local electrograms are considered to display higher frequency components.^4^ These analyses were performed during sinus rhythm. In our case, we gradually increased the cutoff frequency on the emphasis map, which enabled clear delineation of the SVC-RA junction at 350 Hz during AF (Figure 1). The previous findings proved valuable even during AF and were instrumental in accurately identifying the SVC-RA junction. Interestingly, during mapping in sinus rhythm, the frequency in the region corresponding to the RA was 489 Hz, which was higher than that observed during AF (Figure 3). A cutoff frequency of 400 Hz on the emphasis map enabled clear visualization of the SVC-RA junction during sinus rhythm. This finding suggests that the cutoff value for frequency may vary depending on the rhythm, highlighting the importance of further investigation in a larger number of cases.

From the perspective of human embryology, the RA develops through incorporating the sinus venosus into the primitive atrium, resulting in the development of the RA and SVC.^8^ As a result, the structural organization of the SVC and the RA differs, and the myocardium of the SVC is thinner than that of the RA. Embryologic differences are thought to have contributed to identifying the SVC-RA junction through variations in the local electrogram frequency.

## Conclusion

The emphasis map, incorporating a peak frequency annotation algorithm, was useful for identifying the SVC-RA junction even during AF, enabling a successful SVC isolation for an IRAF without causing SNI.

## Disclosures

H.M. received lecture fees from Biosense Webster Japan and Boston Scientific Japan. Our department received grant support from Boston Scientific Japan and Abbott Medical Japan. No other authors have conflicts of interest to disclose.
